# Optimizing Quantum Classification Algorithms on Classical Benchmark Datasets

**DOI:** 10.3390/e25060860

**Published:** 2023-05-27

**Authors:** Manuel John, Julian Schuhmacher, Panagiotis Barkoutsos, Ivano Tavernelli, Francesco Tacchino

**Affiliations:** 1 IBM Quantum, IBM Research Europe—Zurich, 8803 Rüschlikon, Switzerland; 2 Institute for Theoretical Physics, ETH Zürich, 8093 Zurich, Switzerland

**Keywords:** quantum machine learning, quantum classification algorithms, quantum kernel methods

## Abstract

The discovery of quantum algorithms offering provable advantages over the best known classical alternatives, together with the parallel ongoing revolution brought about by classical artificial intelligence, motivates a search for applications of quantum information processing methods to machine learning. Among several proposals in this domain, quantum kernel methods have emerged as particularly promising candidates. However, while some rigorous speedups on certain highly specific problems have been formally proven, only empirical proof-of-principle results have been reported so far for real-world datasets. Moreover, no systematic procedure is known, in general, to fine tune and optimize the performances of kernel-based quantum classification algorithms. At the same time, certain limitations such as kernel concentration effects—hindering the trainability of quantum classifiers—have also been recently pointed out. In this work, we propose several general-purpose optimization methods and best practices designed to enhance the practical usefulness of fidelity-based quantum classification algorithms. Specifically, we first describe a data pre-processing strategy that, by preserving the relevant relationships between data points when processed through quantum feature maps, substantially alleviates the effect of kernel concentration on structured datasets. We also introduce a classical post-processing method that, based on standard fidelity measures estimated on a quantum processor, yields non-linear decision boundaries in the feature Hilbert space, thus achieving the quantum counterpart of the radial basis functions technique that is widely employed in classical kernel methods. Finally, we apply the so-called quantum metric learning protocol to engineer and adjust trainable quantum embeddings, demonstrating substantial performance improvements on several paradigmatic real-world classification tasks.

## 1. Introduction

Machine learning (ML) algorithms are ubiquitous in today’s world. These techniques leverage the natural ability of computers to sieve through vast amounts of data with the aim of revealing the underlying patterns and to accomplish a wide range of tasks, such as image classification, automated generation of text and images or, more generally, decision making.

Quantum computers implement a novel information processing paradigm and may provide an alternative platform for executing machine learning algorithms, an approach known as Quantum Machine Learning (QML) [1,2]. The potential for using quantum computation in machine learning is essentially two-fold. On the one hand, one could, for example, leverage results from quantum optimization or quantum linear algebra [3,4,5,6] to increase the training efficiency of classical ML models [7,8]. On the other hand, native quantum models, such as quantum neural networks [9,10,11,12,13,14,15,16,17], could be engineered to directly carry out specific learning tasks on classical or quantum data and to analyze correlations that are hard to describe or capture classically [1].

Quantum kernel methods applying quantum feature maps naturally emerge from the second line of research. Here, classical input feature vectors are mapped to high-dimensional Hilbert spaces realized with feature-dependent preparation of quantum states [18,19,20]. Once such a quantum embedding is realized, a decision rule to carry out the desired classification task can be obtained directly from the fidelity between the encoded feature vectors [20] or by passing the resulting quantum kernel to a classical support vector machine [18,19]. Soon after the introduction of quantum kernel methods, it was shown that quantum kernel methods, equipped with the right quantum feature maps, can solve certain specifically designed problems more efficiently than any known classical counterpart [21], thus motivating a large body of research aimed at finding similar advantages in more generic and applied contexts [22,23,24], including for the following: data analysis for high-energy physics [25,26,27], quantum phase classification [28], fraud detection [29] and virtual screening for drug discovery [30]. While some promising examples were identified [27,30], only proof-of-principle results have been achieved so far, mostly based on empirical considerations. Moreover, only selected applications may be viable in the near future due to the presence of hardware noise, although several known error suppression and mitigation strategies [31,32,33,34,35] could be employed, and have, in fact, already been tested in the context of QML [12,36].

At the same time, it has recently become clear that the high expressivity of parameterized quantum circuits, together with other properties, such as entangling power or the structure of certain cost functions, can have unintended consequences. In fact, this not only hinders the trainability of variational quantum models—leading to the so-called barren plateau phenomenon [37,38,39]—but also has a negative impact on the capabilities of quantum kernel methods [23,40]. Some of the latest theoretical advancements in the literature have addressed precisely this class of problems: for example, both Kübler et al. [40] and Huang et al. [23] proposed projection-based approaches as well as ways to incorporate inductive biases, i.e., constraints on the range of representable functions. In parallel, Shaydulin et al. [41] proposed a strategy to tune the bandwidth of quantum kernels, which was shown to have effects on generalization performances [42]. However, the exponential concentration of the kernel values, due to high expressivity, entanglement, global measurements, and noise, prevents, in general, the application of fidelity and projection-based quantum kernels to higher numbers of qubits [43].

In this work, we discuss best practices to reduce the impact of known limitations of quantum kernel methods and we explore different general-purpose strategies to systematically enhance the performances of quantum classification algorithms, based on quantum feature maps, with a specific focus on paradigmatic real-world datasets. First, we propose a strategy to alleviate the problem of the exponential concentration in the presence of structured datasets. Our approach is related to the one described in Reference [41], but, instead of global rescaling of the input features, implied by tuning the kernel bandwidth, we employ a separate scaling factor for each feature. In practice, we identify a *domain of rotations* and normalize the input features so as to ensure that the arguments of each parameterized quantum gate in a feature map do not exceed a predefined range (e.g., [−π,π]). As a second step, we describe a classical post-processing procedure that, starting from the usual fidelity measurements between encoded quantum feature vectors, effectively engineers a continuous nearest-neighbor classification rule, and, therefore, enables non-linear decision boundaries in the Hilbert space. This extends the basic notion of quantum kernel and fidelity-based classifiers which, in the standard formulation, only make use of linear separating hyperplanes. Finally, we explore the concept of trainable quantum feature maps, originally introduced for some specific examples by Lloyd et al. [20] and Glick et al. [44]. In this case, we follow the intuition that a generic quantum feature map may not perform well across multiple datasets originating from a wide range of application domains, but should rather be, at least to a certain degree, tailored to the problem. We benchmark this procedure, known as quantum metric learning or quantum kernel alignment, on a collection of paradigmatic datasets of practical relevance.

The remainder of the paper is structured as follows. In Section 2, we introduce some basic concepts related to quantum classification and quantum kernel methods (Section 2.1). We describe the specific quantum algorithms employed in our work (Section 2.2 and Section 2.3) and we provide information about the datasets considered in our numerical experiments (Section 2.4). We present our results in Section 3, while a discussion of their implications and some concluding remarks are contained in Section 4.

## 2. Materials and Methods

### 2.1. Quantum Classification Algorithms and Quantum Embeddings

Classification algorithms from the family of kernel methods rely on a function, called a *kernel*, that quantifies the similarity between data vectors $x_{i}$. For binary classification, the kernel function embeds the data vector into a high-dimensional feature space, where the two classes (ideally) become linearly separable. The success of classical kernel methods stems from the so-called *kernel trick*, which allows one to evaluate the kernel function without explicitly mapping the data to the high-dimensional feature space. The widely used radial basis function (RBF) kernel,

(1) $$K\left(x_{i},x_{j}\right)=\exp\left(-\gamma {∥x_{i}-x_{j}∥}^{2}\right)\;,$$

is an example for which the effective feature space would be infinitely dimensional [45]. However, the kernel itself can be evaluated efficiently.

Quantum computers provide an alternative platform to implement kernel methods, since they provide an efficient way to access high-dimensional Hilbert spaces into which classical data can be embedded [20]. A feature vector x can be mapped into the space of *n*-qubit quantum states by using the entries of x as arguments of a parameterized quantum circuit U(x) [18,19]. We denote the quantum state prepared by applying such a parameterized unitary to the zero state as

(2) |x〉=U(x)|0〉.

The unitary U(x) is often referred to as the quantum feature map or quantum embedding. The potential of using such a method originates from the fact that, in general, quantum embeddings cannot be efficiently simulated with classical computers [18,21]. This is a necessary, albeit not sufficient, condition for achieving quantum advantage with quantum kernel methods.

**Figure 1:**
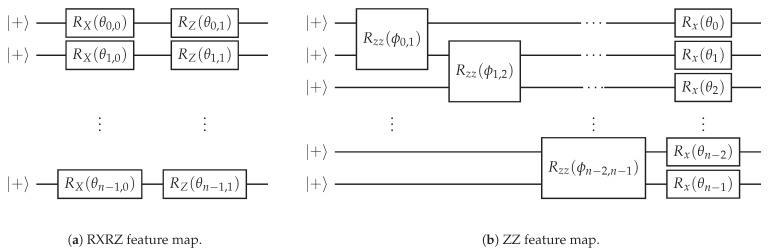
(**a**) One layer of the RXRZ feature map, which produces a classically simulatable output but serves as a reference for the ZZ embedding in (**b**). We optionally apply *L* layers of the depicted gates.

In Figure 1, we present the two feature maps that we apply in our study. The first one, denoted as *RXRZ embedding*, encodes the input features in a layer of single-qubit $R_{X}$ rotations, followed by a layer of $R_{Z}$ rotations (see Figure 1a). Here, with $R_{K}$, K∈{X,Y,Z} we denote the standard Pauli rotation gates. The basic building block shown in Figure 1a can be repeated a number *L* of times, hence producing a *L*-layer version of the feature map. If we choose the parameters in the circuit proportional to the entries $x_{i}$ of the data vector x (i.e., $\theta_{i,\{0,1\}}$∼$x_{i}$) the required number of qubits corresponds to the dimension of the classical data vector. The concrete relation between the parameters and the input features is specified in Section 3.

The second feature map, denoted as *ZZ embedding*, and inspired by similar popular proposals in the literature [18,30,46], is illustrated in Figure 1b. Compared to the RXRZ embedding it additionally contains entangling operators between the qubits in order to capture correlations in the input features. As in the RXRZ case, the ZZ feature map can also be repeated for *L* layers. We choose the parameters of the two qubit $R_{ZZ}$ gates proportional to the product of feature values (i.e., $\phi_{i,j}$∼$x_{i}x_{j}$). The parameters of the subsequent layer of $R_{X}$ rotations are proportional to a single feature value, identical to the RXRZ embedding. The concrete relation between the parameters and the input features is specified in Section 3.

In the space of *n*-qubit quantum states a natural choice for the kernel function is the overlap between two embedded feature vectors $x_{i}$ and $x_{j}$,

(3) $$K\left(x_{i},x_{j}\right)={\left|\langle x_{i}|x_{j}\rangle \right|}^{2}\;.$$

This quantity, also called *fidelity*, can be evaluated on a quantum computer by means of, for example, the so-called SWAP test [47], or the inversion test [18].

### 2.2. Quantum Fidelity and RBF Fidelity Classifiers

**Figure 2:**
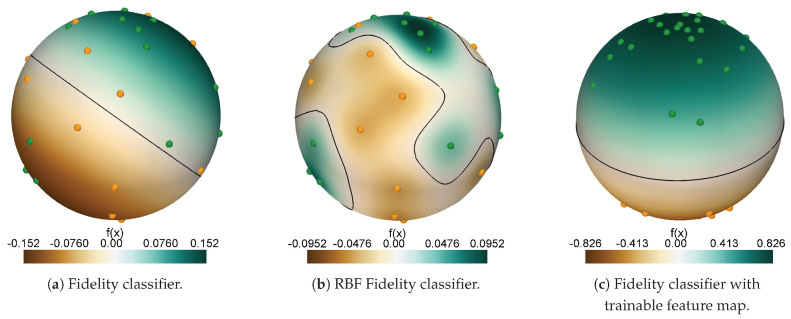
Illustration of a prototypical classification with (**a**) the fidelity classifier, (**b**) the RBF fidelity classifier (with γ=50), and (**c**) the fidelity classifier with trainable feature map. (**a**,**b**) visualize the respective decision functions based on two classes of 25 randomly sampled points on the Bloch sphere. (**c**) shows the ideal outcome of quantum metric learning. The samples of the two embedded classes are mapped to opposing poles of the Bloch sphere. The black line corresponds to the decision boundary, where the decision function evaluates to zero.

For binary classification between two classes A and B, an intuitive method to determine the class label of a new data point x is the fidelity classifier [20]. Given access to reference data points belonging to the two classes (i.e., a training set), we calculate the average fidelity of |x〉 with the embedded data points from classes A and B, denoted as {|a〉} and {|b〉}, respectively. More concretely, the decision function of the fidelity classifier can be written as

(4) $$f\left(x\right)=\frac{1}{M_{A}}\sum_{a\in A}{\left|\langle x|a\rangle \right|}^{2}-\frac{1}{M_{B}}\sum_{b\in B}{\left|\langle x|b\rangle \right|}^{2}\;,$$

where $M_{A}$ is the number of reference points belonging to class A and $M_{B}$ is the number of reference points in class B. If the decision function evaluates to a value f(x)>0 the data point x is assigned to class A, and vice versa if f(x)<0. For this classifier, the corresponding hyperplane separating the two classes is linear in the Hilbert space [20]. In Figure 2a, we visualize the hyperplane obtained with the fidelity classifier for the binary classification between two classes of pure quantum states randomly chosen on the single-qubit Bloch sphere (both the states and the corresponding classes are selected/assigned randomly in this example).

To go beyond linear decision boundaries in the Hilbert space, we propose a classifier based on the following kernel

(5) $$K\left(x_{i},x_{j}\right)=e^{-\gamma (1-{\left|\langle x_{i}|x_{j}\rangle \right|}^{2})}\;,$$

inspired by the classical RBF kernel presented in Equation (1). Here γ is a tunable hyperparameter. The classifier obtained with the decision function

(6) $$f\left(x\right)=\frac{1}{M_{A}}\sum_{a\in A}e^{-\gamma (1-{\left|\langle x|a\rangle \right|}^{2})}-\frac{1}{M_{B}}\sum_{b\in B}e^{-\gamma (1-{\left|\langle x|b\rangle \right|}^{2})}\;,$$

is denoted as *RBF fidelity classifier* in the following. Evaluating the decision function requires the same amount of quantum resources as for the fidelity classifier, since the exponentiation is a simple post-processing of the fidelity values. We apply the same decision rule as for the fidelity classifier. Using the RBF fidelity for classification only considers data points within a neighborhood of |x〉, where the range of this neighborhood is determined by γ, and the fidelity is used as a distance metric. Since this approach is more flexible than the fidelity classifier, we expect that the RBF fidelity classifier will offer better classification performance. In Figure 2b, we visualize the non-linear hyperplane obtained with the RBF fidelity classifier for the same binary classification as for the fidelity classifier above.

### 2.3. Quantum Metric Learning

It has been shown in References [20,44] that adding trainable parts to the quantum feature map can lead to an improvement in the classification performance of quantum kernel and fidelity-based models. Instead of manipulating the separating hyperplane (as in the case of the RBF classifier presented in Section 2.2 above), these approaches manipulate the feature map by tailoring it to the considered classification task. Following the approach presented in Reference [20], called *quantum metric learning*, we can introduce trainable parameters $\alpha_{i,j}$ and $\beta_{i}$ to the gates in the ZZ embedding,

(7) $${\tilde{\phi }}_{i,j}=\phi_{i,j}+\alpha_{i,j}\;{\tilde{\theta }}_{i}=\theta_{i}+\beta_{i}\;.$$

Optimizing these parameters changes the form of the embedding of the data points in the Hilbert space, and, therefore, effectively modifies the relations between them.

The cost function used for the optimization of the parameters is the empirical risk computed for the decision function $f_{\alpha ,\beta }\left(x\right)$,

(8) $$I\left[f_{\alpha ,\beta }\right]=-\frac{1}{M}\sum_{m=1}^{M}L\left(f_{\alpha ,\beta }\left(x_{m}\right),y_{m}\right)\;,$$

where $L(f_{\alpha ,\beta }\left(x\right),y)$ is the loss function, x are the data samples, *y* the corresponding labels, the subscript (α,β) denotes the dependence on the trainable parameters and *M* is the total number of available data samples in the reference data set. Using the fidelity classifier and L(f(x),y)=f(x)·y as the loss function, the parameters are then optimized such that data points belonging to the same class are mapped to close regions in the Hilbert space, and data points belonging to different classes are mapped to distant regions in the Hilbert space [20]. In Figure 2c, we illustrate the desired effect of optimizing the feature map. Ideally, optimizing the embedding parameters maps the two classes to opposing poles of the Bloch sphere. This allows for accurate classification using the linear hyperplane of the fidelity classifier.

### 2.4. Datasets

We evaluated the performance of the quantum classifiers introduced above on six different datasets, representing paradigmatic classification tasks from a broad range of application domains. The datasets and their characteristics are listed in Table 1.

## 3. Results

### 3.1. Pre-Processing

Before evaluating the performance of the classifiers on the different datasets introduced above, we first present a study on different pre-processing strategies for the data. After an initial cleaning of the datasets (i.e., removing duplicates, defective samples, etc.) we performed the following pre-processing steps. First, we standardized the data in each feature $x_{i}$, by subtracting its mean $\mu_{i}$ and dividing by its standard deviation $\sigma_{i}$

(9) $$x_{i}\mapsto \frac{x_{i}-\mu_{i}}{\sigma_{i}}\;.$$

Both the mean and standard deviation were calculated over the training data set, and the respective transformation was then also applied to the test data set. The standardized features were then projected to an *n*-dimensional feature vector using principal component analysis (PCA) [55]. In the literature, these two steps often represent the full pre-processing pipeline. However, consistent with the observations made in Reference [41], we demonstrated that subsequent scaling or normalization of the features has a beneficial effect on the performance of the resulting classifier. We compared the following three cases: The first option directly uses the principal components as input to the feature maps. The second option applies a global scaling factor λ to each principal component $x_{i}\mapsto \lambda x_{i}$, as in Reference [41]. The optimal scaling factor is determined via cross-validation. As a third option, we propose normalizing each feature to a fixed interval [a,b] with min–max normalization

(10) $$x_{i}\mapsto a+\frac{\left(x_{i}-x_{i,\min}\right)\left(b-a\right)}{x_{i,\max}-x_{i,\min}}\;,$$

where $x_{i,\min}$ and $x_{i,\max}$ are the minimal and maximal values of feature $x_{i}$ over the training set, respectively. The motivation for the latter was to map all features to a suitable *domain of rotations*. This ensures that all arguments given as inputs to a parameterized feature map lie within the range [−π,π] (or alternatives thereof), such that the mapping becomes injective. In practice, we achieve mapping to the domain of rotations by normalizing each feature $x_{i}$ to the interval [0,1], and by selecting the parameters in the feature maps accordingly. For the RXRZ feature map, presented in Figure 1a, we chose parameters $\theta_{i,0}=\pi x_{i}$ and $\theta_{i,1}=2\pi x_{i}$, as this guaranteed that the arguments to the $R_{X}$ and $R_{Z}$ rotations were in [0,2π]. For the ZZ feature map, introduced in Figure 1b, we chose the parameters $\phi_{ij}=2\pi x_{i}x_{j}$ and $\theta_{i}=\pi x_{i}$, which guaranteed that the arguments of the two-qubit rotations were in [0,2π], and the arguments of the single-qubit rotations were in [0,π]. The same expressions for the θ and ϕ parameters, including the π or 2π factors, were also used for the other pre-processing strategies.

**Figure 3:**
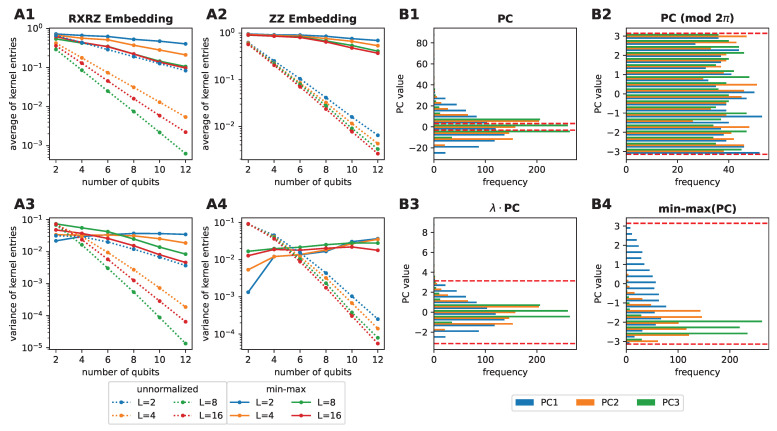
Illustration of the exponential concentration of the kernel values (panels (**A1**–**A4**)), and the effect of the pre-processing on the distributions of the input features (panels (**B1**–**B4**)). In panels (**A1**–**A4**), we used L=2,4,8,16 layers of the RXRZ or ZZ feature maps to embed 800 data samples in the fMNIST dataset. Panels (**B1**–**B4**) show the distribution of the first three principal components (PCs) of the considered data samples in the fMNIST data set, subject to different pre-processing strategies. The interval [−π,π] is highlighted with red dotted lines. (**B1**) “Raw” principal components. (**B2**) “Raw” principal components, periodically mapped to the highlighted interval. (**B3**) Principal components scaled with λ=0.1. (**B4**) Principal components normalized to the highlighted interval.

To compare the different pre-processing pipelines, we conducted the following experiment, inspired by a similar study performed in Reference [43]. As a starting point, we calculated the kernel matrix $K_{ij}=K\left(x_{i},x_{j}\right)={\left|\langle x_{i}x_{j}\rangle \right|}^{2}$ of 800 data samples in the fMNIST dataset for increasing numbers of features *n* (and, hence, also increasing number of qubits), and for increasing numbers of layers *L* in the feature map. We then evaluated the average and the variance across the entries in the resulting kernel matrix. The corresponding results are displayed in Figure 3A1–A4. The average and variance are shown for the kernels produced by applying the pre-processing with a final normalization step (solid lines) and without a final normalization step (dotted lines), using the RXRZ embedding (panels A1 and A3), or the ZZ embedding (panels A2 and A4). In all cases, the average and the variance decayed exponentially if no normalization was applied. This agreed with the results in Reference [43], where the authors demonstrated that increasing the expressivity of a feature map, and employing global fidelity measurements on uniformly sampled inputs, led to exponential concentration of the resulting kernel entries. However, the exponential concentration, as well as the exponential decay of the average kernel entries, are less pronounced when normalization to the domain of rotations is applied. For the ZZ feature map, we even observed a modest increase in the variance when increasing the number of features.

In Figure 3B1–B4 we illustrate an intuitive explanation of the effect of the pre-processing. The panels show the distribution of the first three principal components subjected to the different pre-processing strategies. As a visual guideline, the interval [−π,π] is highlighted with the red dashed lines. The distributions of the non-normalized principal components mostly lay outside the highlighted interval (panel B1). Periodic mapping (such as that enforced by Pauli rotation gates) of these distributions into the highlighted interval led to distributions resembling a uniform distribution (panel B2). The original structure of the dataset was, thus, lost and the embedded data points hardly represented the original dataset faithfully. In fact, under these conditions the distribution of the input features tended to quickly become close to uniform over one period of the encoding Pauli rotations, such that some of the concentration hypotheses made in Reference [43], particularly for the case of global fidelity kernels, were essentially met. Applying a scaling factor λ=0.1 to all principal components (the approach used in Reference [41]) led, instead, to the distributions displayed in panel B3. We observed that the most important regions of the distribution now lay within the highlighted interval, such that most of the structure would be preserved, even under periodicity. However, only our proposed min–max inspired option, namely the normalization to the domain of rotations, fully restricted each principal component individually to the interval [−π,π] (panel B4). Compared to the distribution in B1, a periodic mapping to the highlighted interval preserved the distribution from B4, as all values were already in the correct domain. In summary, careful pre-processing represents a fast, direct and problem-agnostic method to mitigate kernel concentration effects leveraging the intrinsic data distribution, whenever that is present.

In the next section we describe the effects of the pre-processing on the performance of the resulting classifier.

### 3.2. Classification

To evaluate the performance of the classifiers under study, we considered different standard metrics, namely the balanced accuracy, the receiver operator characteristic area under curve (ROC AUC) and the F1 score. The detailed definitions of these scoring methods are given in Appendix A. All the scores presented in the following were evaluated by averaging over 100 train–test splits (80–20%) of a specific dataset. In addition, if the scaling factor or RBF γ hyperparameters were to be determined, we performed five-fold cross validation on the train set of a split. The scaling factor was chosen from the interval $\left[10^{-3},1\right]$ and the γ parameter from $\left[10^{-5},10^{3}\right]$.

**Figure 4:**
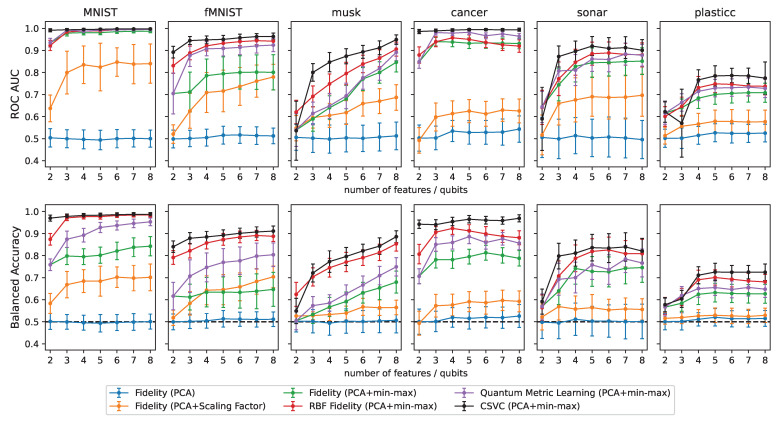
ROC–AUC and balanced accuracy for different datasets and increasing number of features. The blue, orange and green lines show the performance of the fidelity classifier resulting from the pre-processing of the input data without normalization, scaling with an optimized scaling factor, or with normalization, respectively. The red lines show the performance of the RBF fidelity classifier (using the same pre-processing steps as for the green line). The purple lines show the performance of the fidelity classifier with a trainable feature map (using the same pre-processing steps as for the green line). For all classifiers, the ZZ feature map (or its trainable version), was used for the embedding of the data points. The black lines show the performance of the classical support vector classifier. In the bottom row, the black dashed lines show the balanced accuracy achieved by a random classifier.

The ROC–AUC score and the balanced accuracy of all studied classifiers applied to the datasets in Table 1 are shown in Figure 4. All classifiers were built via the embedding of the pre-processed input features with the ZZ feature map. The blue, orange and green lines show the performances of the fidelity classifier resulting from the pre-processing of the data without normalization, scaling with an optimized scaling factor, or with normalization, respectively. For all datasets, applying no normalization to the principal components, resulted in classifiers that had, essentially, the same performance as a random classifier (value of 0.5 for both performance metrics). Applying a joint scaling factor to all principal components, led to considerable improvement in the performances of the classifiers for most datasets. However, in almost all cases, our proposed normalization to the domain of rotations (which effectively corresponds to applying an individual scaling factor to each feature), led to substantial improvements over the other two pre-processing strategies. For the rest of the investigation, we, therefore, applied the normalization to the domain of rotations as the last step of the pre-processing pipeline.

As a next step, we look at the performance of the proposed RBF fidelity classifier (red lines in Figure 4). The effects of the simple post-processing of the fidelities, required to build the RBF classifier, were noticeable for all datasets. In fact, the RBF fidelity classifier performed better than the standard fidelity classifier (green lines) in almost all cases, with significant improvements for the fMNIST, cancer, and sonar datasets. Note that the cross-validation to find the best hyperparameter for the RBF fidelity classifier did not lead to an increase in the required quantum resources. The fidelity kernel only had to be evaluated once, and the cross-validation could then be performed classically.

Finally, the performance of the fidelity classifier with a trainable feature map, is depicted in Figure 4 with the purple lines. The approach led to noticeable improvements over the standard fidelity classifier for the MNIST, fMNIST, musk and cancer datasets. The effect was less pronounced for the remaining datasets. Nonetheless, the results still showcase the potential of tailoring the feature map to the considered classification task. However, compared to the RBF fidelity classifier, the trainable fidelity classifier performed worse in most of the cases.

For completeness, we also provide, in Figure 4, the results obtained for all datasets with a classical Support Vector Classifier (CSVC) featuring either a linear or an RBF kernel (where all hyperparameters were chosen by five-fold cross-validation). While the performances of the CSVC still represented an upper bound, a quantum classifier with our proposed improvements often achieved comparable classification scores. Overall, these findings suggest that our techniques could become part of a toolbox aimed at maximising empirical performances of quantum classification algorithms, and, hence, provide opportunities for quantum advantage, when scaling up the size of practical applications.

## 4. Discussion and Conclusions

The results presented in this study demonstrate the importance of proper data pre-processing in quantum machine learning, specifically in the context of quantum kernel methods for classification. Our experiments, conducted on a variety of structured real-world datasets, showed that the absence of a suitable normalization procedure could lead to essentially random quantum embeddings, characterized by a loss of the relationships between the data, exponential concentration in the kernel values and, most notably, poor classification performance. We illustrated the effect of different feature normalization strategies using various scaling methods, and demonstrated that our proposed normalization approach consistently led to improved performance across all tested datasets and all numbers of principal components. It is also worth mentioning that, while an optimized global scaling factor controlling the kernel bandwidth [41] can, in general, only be found through cross-validation, requiring several kernel evaluations for multiple train–test splits, the normalization approach is defined solely by the input data, and can, therefore, be applied without any substantial computational overhead.

We also investigated the effect of non-linear post-processing of the fidelity quantum kernel entries through exponentiation, yielding an original and effective RBF-like quantum kernel, and of quantum metric learning across a broad range of application domains. In both cases, for a representative collection of datasets, significant improvements, in terms of classification performances with respect to standard quantum methods, were observed. As a result, we conclude that these approaches constitute an effective and relatively inexpensive toolbox that could be applied in many realistic scenarios to systematically improve the performances of quantum classification algorithms.

## Figures and Tables

**Table 1:** Datasets used in this study and their properties after cleaning the data (removing duplicates, defective samples, etc.).

Dataset	# Features	# Positives	# Negatives	Source	Description
MNIST	28 × 28	500	500	[48]	Grayscale images of hand-written digits (0’s vs. 9’s)
fMNIST	28 × 28	500	500	[49]	Grayscale images of clothing (T-shirts vs. dresses)
musk	166	207	269	[50,51]	Molecules occurring in different conformations (musk vs. non-musk)
sonar	60	97	111	[51,52]	Sonar signals (bounced off a metal cylinder vs. a roughly cylindrical rock)
cancer	30	212	357	[53]	Characteristics of breast cancer tumors (benign vs. malignant)
plasticc	67	500	500	[54]	Photometric LSST Astronomical Time-series Classification Challenge dataset. Pre-processed by [22] (type II vs. Ia supernovae)

## Data Availability

The data presented in this study are available from the corresponding author upon reasonable request.

## Appendix A. Performance Metrics

Most scoring methods can be derived from a so-called confusion matrix. For binary classification, such a confusion matrix summarizes the classification performance with four values:

- true positives (TP)—number of positive samples classified as positive
- false positives (FP)—number of negative samples classified as positive
- true negatives (TN)—number of negative samples classified as negative
- false negatives (FN)—number of positive samples classified as negative

For clarity, we also introduce the total number of positive samples P and the total number of negative samples N.

The most intuitive method to evaluate performance is to look at how many samples out of the entire test set were correctly classified. Accuracy is then defined as

(A1) $$a=\frac{\mathrm{TP}+\mathrm{TN}}{\mathrm{TP}+\mathrm{FP}+\mathrm{TN}+\mathrm{FN}}=\frac{\mathrm{TP}+\mathrm{TN}}{P+N}\;.$$

This score is well suited for those cases in which the dataset is balanced (same number of positives and negatives) and if both classes are of equal importance. However, it can become a misleading figure of merit on imbalanced datasets. As an alternative, balanced definition of accuracy takes into account an average over each class. For binary classification, we define the accuracy on the positive samples as the true positive rate TPR=TP/P and the accuracy on the negative samples as the true negative rate TNR=TN/N. The balanced accuracy can then be defined as

(A2) $$a_{\mathrm{balanced}}=\frac{\mathrm{TPR}+\mathrm{TNR}}{2}\;.$$

The Receiver Operator Characteristic Area Under Curve (ROC–AUC) is a metric that, in addition to classification of performances, also conveys information about the robustness of the model. In a nutshell, the ROC follows the true positive rate and the false positive rate while continuously moving the decision boundary of the classifier from an extreme condition where all data are classified as negative to the opposite scenario, where all data are considered positive. The area under the ROC curve is then a measure of the overall quality of the classifier. A completely random classifier on a balanced dataset achieves an ROC–AUC of 0.5.

The F1 score is the harmonic mean of the precision (how many out of all positively classified samples are positive) and the true positive rate (how many out of the positive samples were classified as positive). Ideally, our classifier would achieve a high score on both the precision and the TPR—In fact, an ideal model only classifies positive data points as positives and at the same time finds all the positives. Typically there is a trade-off between the two requirements, which is summarized by the F1 score

(A3) $$F1=\frac{2\mathrm{TP}}{\left(\mathrm{TP}+\mathrm{FP})+(\mathrm{TP}+\mathrm{FN}\right)}$$

As this is a robust scoring method, we used it to evaluate the cross-validation on all datasets.
